# Supplementary material for: Costs and cost-effectiveness of a comprehensive tuberculosis case finding strategy in Zambia
Source: PLoS One. 2021 Sep 9;16(9):e0256531. doi: 10.1371/journal.pone.0256531 (PMC8428570; doi:10.1371/journal.pone.0256531)
Supplement: S1 File — (DOCX) [file pone.0256531.s001.docx]

**Supporting Information**

**Figure S1. Quarterly program statistics and unit costs (July 2017 – December 2018)**

We collected cost and service volume data on a quarterly basis. The graph shows the variation in service volume and unit cost per service and outcome from July 2017 to December 2018. For example, service volumes of population enrollment and CXR test (which was used as screening tool in this program) were increasing until April-June 2018 and decreasing after, potentially due to increasing public awareness on the campaign and improving technical efficiency in screening over time in the beginning. There was also a cholera outbreak during the first six months (Jul-Dec, 2017), which delayed the initiation of community-based CXR. Also, the truck on which mobile chest X-ray was performed broke down during the last six months (Jul-Dec, 2018). Therefore, facility-based screening was mainly conducted during the first and last six months periods, reflecting lower service volume during the time. On the other hand, variation of the number of TB cases diagnosed by CXR test (45-104 yields quarterly) and the service volume and yields of Xpert test (about 1000 tests and 200 yields quarterly) were relatively constant over time. This may be because the CXR truck was moving to each different village per week for screening and thus the number of TB cases diagnosed by CXR or Xpert reflect general TB prevalence in the district.

In terms of cost per service, in the first two quarters, both Xpert cost (quarterly cost about $30,000) and service volume (1,110) were much greater than CXR cost ($3,500) and service volume (463), resulting a higher Xpert unit cost than CXR unit cost ($27 vs $7). This is because of the limited community-based screening operation due to the cholera outbreak. During the last two quarters, however, the total Xpert cost was lower than total Xray cost ($10,000 vs $30,000), while the service volume of Xpert was similar to Xray (1,179 vs 1,293), resulting a higher CXR unit cost than Xpert unit cost ($26 vs $10). Quarterly variation in cost per service were incorporated into the model as uncertainty ranges. (See Tables 1 and 2 in the main text.)

| - Service volumes per quarter | - Cost per service |
| --- | --- |
|  |  |
| - Number of outcomes per quarter | - Cost per outcome |
|  |  |

**Table S1. Quarterly program costing and unit costs estimations (July 2017 – December 2018)**

| **June-December 2017** | | | | | | | | | | | | | | |
| --- | --- | --- | --- | --- | --- | --- | --- | --- | --- | --- | --- | --- | --- | --- |
| **Resource categories** | **Total cost** | **%** | **Community Sensitization** | | | **Training** | | **Screening** | | **X-ray** | | **Xpert** | | **Treatment** |
| Human resources | $31,379 | 24% | $1,374 | | | $2,709 | | $2,859 | | $3,190 | | $2,038 | | $810 |
| Capital cost | $61,328 | 47% | $1,425 | | | $335 | | $16,930 | | $3,463 | | $39,175 | | $0 |
| Recurrent costs | $34,724 | 27% | $2,880 | | | $0 | | $1,055 | | $151 | | $20,588 | | $0 |
| Overhead costs | $1,706 | 1% | $79 | | | $156 | | $76 | | $183 | | $117 | | $37 |
| **Total activity costs** | $129,137 | 100% | $5,758 | | | $3,200 | | $20,921 | | $6,988 | | $61,918 | | $848 |
| **Incremental costs for yield** | 0 | 0% | $0.00 | | | $0.00 | | $29,878 | | $36,866 | | $98,784 | | $99,631 |
| **Number of beneficiaries** | Total # | % | **Cost per activity** | | | | | | | | | | | |
| Population enrollment | 4768 | 100% | $1.21 | | | $0.67 | | $4.39 | | $0 | | $0 | | $0 |
| X-ray test | 927 | 19% | $0 | | | $0 | | $0 | | $7.54 | | $0 | | $0 |
| X-pert test | 2220 | 47% | $0 | | | $0 | | $0 | | $0 | | $27.89 | | $0 |
| treatment | 302 | 6% | $0 | | | $0 | | $0 | | $0 | | $0 | | $2.81 |
| **Number of yields** | Total # | % | **Cost per yield** | | | | | | | | | | | |
| Screened as TB suspects | 2406 | 50% | NA | | | | | $12.42 | | $0 | | $0 | | $0 |
| Diagnosed as TB cases by X-ray | 90 | 2% |  |  |  |  |  | $0 | | $409.62 | | $0 | | $0 |
| Diagnosed as TB cases by Xpert | 194 | 4% |  |  |  |  |  | $0 | | $0 | | $509.19 | | $0 |
| Treatment initiated | 302 | 6% |  |  |  |  |  | $0 | | $0 | | $0 | | $329.91 |
| **January-March 2018** | | | | | | | | | | | | | | |
| **Resource categories** | **Total cost** | **%** | **Community Sensitization** | | | **Training** | | **Screening** | | **X-ray** | | **Xpert** | | **Treatment** |
| Human resources | $11,581 | 19% | $1,134 | | | $2,374 | | $2,738 | | $1,580 | | $2,830 | | $1,063 |
| Capital cost | $7,359 | 12% | $1,281 | | | $51 | | $5,127 | | $0 | | $900 | | $0 |
| Recurrent costs | $38,310 | 64% | $1,827 | | | $0 | | $929 | | $28,895 | | $6,659 | | $0 |
| Overhead costs | $2,884 | 5% | $331 | | | $693 | | $350 | | $461 | | $826 | | $264 |
| **Total activity costs** | $60,135 | 100% | $4,573 | | | $3,118 | | $9,144 | | $30,936 | | $11,215 | | $1,327 |
| **Incremental costs for yield** | 0 | 0% | $0.00 | | | $0.00 | | $16,835 | | $47,771 | | $58,986 | | $60,313 |
| **Number of beneficiaries** | Total # | % | **Cost per activity** | | | | | | | | | | | |
| Population enrollment | 3346 | 100% | $1.37 | | | $0.93 | | $2.73 | | $0 | | $0 | | $0 |
| X-ray test | 2,709 | 81% | $0 | | | $0 | | $0 | | $11.42 | | $0 | | $0 |
| X-pert test | 718 | 21% | $0 | | | $0 | | $0 | | $0 | | $15.62 | | $0 |
| treatment | 177 | 5% | $0 | | | $0 | | $0 | | $0 | | $0 | | $7.50 |
| **Number of yields** | Total # | % | **Cost per yield** | | | | | | | | | | | |
| Screened as TB suspects | 867 | 26% | NA | | | | | $19.42 | | $0 | | $0 | | $0 |
| Diagnosed as TB cases by X-ray | 83 | 2% |  |  |  |  |  | $0 | | $575.55 | | $0 | | $0 |
| Diagnosed as TB cases by Xpert | 91 | 3% |  |  |  |  |  | $0 | | $0 | | $648.20 | | $0 |
| Treatment initiated | 177 | 5% |  |  |  |  |  | $0 | | $0 | | $0 | | $340.75 |
| **April-June 2018** | | | | | | | | | | | | | | |
| **Resource categories** | **Total cost** | **%** | **Community Sensitization** | | | **Training** | | **Screening** | | **X-ray** | | **Xpert** | | **Treatment** |
| Human resources | $35,611 | 29% | $1,272 | | | $1,863 | | $2,644 | | $3,100 | | $2,455 | | $739 |
| Capital cost | $7,794 | 6% | $402 | | | $0 | | $5,912 | | $0 | | $1,480 | | $0 |
| Recurrent costs | $74,748 | 62% | $1,440 | | | $0 | | $1,461 | | $63,588 | | $7,680 | | $0 |
| Overhead costs | $3,011 | 2% | $113 | | | $165 | | $98 | | $275 | | $218 | | $51 |
| **Total activity costs** | $121,164 | 100% | $3,227 | | | $2,028 | | $10,115 | | $66,963 | | $11,833 | | $790 |
| **Incremental costs for yield** | 0 | 0% | $0.00 | | | $0.00 | | $15,370 | | $82,333 | | $94,166 | | $94,956 |
| **Number of beneficiaries** | Total # | % | **Cost per activity** | | | | | | | | | | | |
| Population enrollment | 6601 | 100% | $0.49 | | | $0.31 | | $1.53 | | $0 | | $0 | | $0 |
| X-ray test | 6,038 | 91% | $0 | | | $0 | | $0 | | $11.09 | | $0 | | 0 |
| X-pert test | 813 | 12% | $0 | | | $0 | | $0 | | $0 | | $14.55 | | 0 |
| treatment | 178 | 3% | $0 | | | $0 | | $0 | | $0 | | $0 | | $4.44 |
| **Number of yields** | Total # | % | **Cost per yield** | | | | | | | | | | | |
| Screened as TB suspects | 1160 | 18% | NA | | | | | $13.25 | | $0 | | $0 | | $0 |
| Diagnosed as TB cases by X-ray | 82 | 1% |  |  |  |  |  | $0 | | $1,004.06 | | $0 | | $0 |
| Diagnosed as TB cases by Xpert | 96 | 1% |  |  |  |  |  | $0 | | $0 | | $980.90 | | $0 |
| Treatment initiated | 178 | 3% |  |  |  |  |  | $0 | | $0 | | $0 | | $533.46 |
| **July-September 2018** | | | | | | | | | | | | | | |
| **Resource categories** | **Total cost** | **%** | **Community Sensitization** | | | **Training** | | **Screening** | | **X-ray** | | **Xpert** | | **Treatment** |
| Human resources | $12,807 | 22% | $1,272 | | | $1,863 | | $2,644 | | $3,467 | | $2,822 | | $739 |
| Capital cost | $7,359 | 12% | $1,281 | | | $51 | | $5,127 | | $0 | | $900 | | $0 |
| Recurrent costs | $38,310 | 64% | $1,827 | | | $0 | | $929 | | $28,895 | | $6,659 | | $0 |
| Overhead costs | $945 | 2% | $108 | | | $158 | | $94 | | $295 | | $240 | | $49 |
| **Total activity costs** | $59,421 | 100% | $4,488 | | | $2,072 | | $8,795 | | $32,657 | | $10,621 | | $788 |
| **Incremental costs for yield** | 0 | 0% | $0.00 | | | $0.00 | | $15,355 | | $48,012 | | $58,633 | | $59,421 |
| **Number of beneficiaries** | Total # | % | **Cost per activity** | | | | | | | | | | | |
| Population enrollment | 3947 | 100% | $1.14 | | | $0.52 | | $2.23 | | $0 | | $0 | | $0 |
| X-ray test | 3,005 | 76% | $0 | | | $0 | | $0 | | $10.87 | | $0 | | $0 |
| X-pert test | 760 | 19% | $0 | | | $0 | | $0 | | $0 | | $13.98 | | $0 |
| treatment | 190 | 5% | $0 | | | $0 | | $0 | | $0 | | $0 | | $4.15 |
| **Number of yields** | Total # | % | **Cost per yield** | | | | | | | | | | | |
| Screened as TB suspects | 998 | 25% | NA | | | | | $15.39 | | $0 | | $0 | | $0 |
| Diagnosed as TB cases by X-ray | 104 | 3% |  |  |  |  |  | $0 | | $461.65 | | $0 | | $0 |
| Diagnosed as TB cases by Xpert | 90 | 2% |  |  |  |  |  | $0 | | $0 | | $651.47 | | $0 |
| Treatment initiated | 190 | 5% |  |  |  |  |  | $0 | | $0 | | $0 | | $312.74 |
| **October-December 2018** | | | | | | | | | | | | | | |
| **Resource categories** | **Total cost** | **%** | **Community Sensitization** | | | **Training** | | **Screening** | | **X-ray** | | **Xpert** | | **Treatment** |
| Human resources | $16,310 | 26% | $1,272 | | | $1,863 | | $3,739 | | $3,905 | | $3,548 | | $1,983 |
| Capital cost | $7,359 | 12% | $1,281 | | | $51 | | $5,127 | | $0 | | $900 | | $0 |
| Recurrent costs | $38,310 | 61% | $1,827 | | | $0 | | $929 | | $28,895 | | $6,659 | | $0 |
| Overhead costs | $1,243 | 2% | $108 | | | $158 | | $187 | | $332 | | $302 | | $155 |
| **Total activity costs** | $63,222 | 100% | $4,488 | | | $2,072 | | $9,983 | | $33,132 | | $11,409 | | $2,138 |
| **Incremental costs for yield** | 0 | 0% | $0.00 | | | $0.00 | | $16,543 | | $49,675 | | $61,084 | | $63,222 |
| **Number of beneficiaries** | Total # | % | **Cost per activity** | | | | | | | | | | | |
| Population enrollment | 1724 | 100% | $2.60 | | | $1.20 | | $5.79 | | $0 | | $0 | | $0 |
| X-ray test | 1,293 | 75% | $0 | | | $0 | | $0 | | $25.62 | | $0 | | $0 |
| X-pert test | 1179 | 68% | $0 | | | $0 | | $0 | | $0 | | $9.68 | | $0 |
| treatment | 158 | 9% | $0 | | | $0 | | $0 | | $0 | | $0 | | $13.53 |
| **Number of yields** | Total # | % | **Cost per yield** | | | | | | | | | | | |
| Screened as TB suspects | 1164 | 68% | NA | | | | | $14.21 | | $0 | | $0 | | $0 |
| Diagnosed as TB cases by X-ray | 158 | 9% |  |  |  |  |  | $0 | | $314.40 | | $0 | | $0 |
| Diagnosed as TB cases by Xpert | 62 | 4% |  |  |  |  |  | $0 | | $0 | | $985.22 | | 0 |
| Treatment initiated | 96 | 6% |  |  |  |  |  | $0 | | $0 | | $0 | | $658.56 |
| **Types of services** | **Average**  **unit cost** | | | **Cost per activity** | | | | | | | | | | |
|  |  |  |  | **Jul-Sep, 2017** | **Oct-Dec, 2017** | | **Jan-Mar 2018** | | **Apr-Jun, 2018** | | **Jul-Sep, 2018** | | **Oct-Dec, 2018** | |
| Population enrollment | $3.33 | | | $4.39 | $4.39 | | $2.73 | | $1.53 | | $2.23 | | $5.79 | |
| X-ray test | $13.31 | | | $7.54 | $7.54 | | $11.42 | | $11.09 | | $10.87 | | $25.62 | |
| X-pert test | $16.34 | | | $27.89 | $27.89 | | $15.62 | | $14.55 | | $13.98 | | $9.68 | |
| Diagnosed as TB cases by X-ray | $553 | | | $409.62 | $409.62 | | $575.55 | | $1,004.06 | | $461.65 | | $314.40 | |
| Diagnosed as TB cases by Xpert | $755 | | | $509.19 | $509.19 | | $648.20 | | $980.90 | | $651.47 | | $985.22 | |
| Treatment initiated | $435 | | | $329.91 | $329.91 | | $340.75 | | $533.46 | | $312.74 | | $658.56 | |

**Table S2. Symptom transition and care seeking model**

We constructed a symptom transition Markov model ^1^ in a population of 1000 simulated individuals under two different conditions: open cohort (entry and exit from the cohort allowed, to maintain a constant population size) and closed cohort (following 1000 individuals from entry until death or TB cure, with no entries allowed). Each model was calibrated to country-specific prevalence and incidence. We defined three symptom levels (asymptomatic, nonspecific, and classic) based on the corresponding probability of diagnostic evaluation for TB (Figure S2, Panel A and B) with the following assumptions: 83% patients who develop incident TB ultimately receive a diagnostic test (assuming a diagnostic sensitivity of 85%), and 93% of patients receiving treatment are cured. We also assumed that 80% of individuals with non-specific symptoms and 100% with classic symptom would initiate treatment based on empiric treatment behavior. First, we calibrated the model parameters to meet the three key constraints in the closed model: 1. The probability of progression is two times that of regression; 2. The lifetime probability of TB self-cure equals that of death in the absence of treatment (Panel C); and 3. The mean duration of the asymptomatic period is 9 months. (Panel D)^2^ Second, in the open model, we calibrated the monthly probability of patients contacting the health system in order to achieve a steady state that resulted in equilibrium values of TB incidence and prevalence that matched WHO estimates for Zambia in 2018. The calibrated results in open/closed models were presented in Figure 2.

| **Model input parameters** | **Values** | **Data source/assumptions** |
| --- | --- | --- |
| Population epidemiology |  |  |
| Prevalence of TB in Zambia yearly (388/100,000) | 0.00388 | 3 |
| Incidence of TB in Zambia per year (346/100,000) | 0.00346 |  |
| Duration of disease | 0.99233 | Prevalence/Incidence |
| Number of TB+ people in model | 1,000 | Assumption |
| Number of people enter with no symptom every year | 1,008 | 1/duration of diseases |
| Number of people enter with no symptom every round (per month) | 83.98 | 1008 / 12 months |
| TB Epidemiology |  |  |
| Probability of having no symptom with TB | 0.2 | 4  Panel (A and B) |
| Probability of having nonspecific symptom with TB | 0.300 |  |
| Probability of having classic symptom with TB | 0.500 |  |
| Programmatic features [Diagnostic algorithms & Empirical treatment] |  |  |
| Probability of receiving diagnostic test among passive contact | 0.826 | 5 |
| Sensitivity of diagnostic tests | 0.850 |  |
| Probability of cure among patients received treatment | 0.930 |  |
| Probability of treatment of asymptomatic patients with negative diagnosis | 0.000 |  |
| Probability of treatment of nonspecific symptom patients with negative diagnosis | 0.800 |  |
| Probability of treatment of classic symptom patients with negative diagnosis | 1.000 |  |
| **Calibrated model outputs** |  |  |
| Proportion of patients having passive contact with health system by symptom levels | | |
| Percent of patient who seek care per month | 0.000 | Calibrate with open system to the extent entry & exit reach equal in numbers at equilibrium (Panel C) |
| Percent of nonspecific symptom patient who seek care per month | 0.200 |  |
| Percent of classic symptom patient who seek care per month | 0.400 |  |
| Monthly Symptom Transition Rate | | |
| Probability of transition from asymptomatic to cure (self-cure) | 0.050 | While satisfying the condition as the probability of progression is 2 times that of regression, calibrate with closed/open system that lifetime probability of TB self-cure equals that of death in the absence of treatment (Panel C) |
| Probability of transition from nonspecific to asymptomatic (regression) | 0.120 |  |
| Probability of transition from asymptomatic to nonspecific (progression) | 0.240 |  |
| Probability of transition from nonspecific to classic (progression) | 0.800 |  |
| Probability of transition from classic to nonspecific (regression) | 0.400 |  |
| Probability of transition from classic to death^a^ | 0.050 |  |

1. We assumed untreated case fatality ratio of 0.5 based on an average estimate based on *Monthly Untreated TB Mortality for HIV positive/negative and TB negative/positive in the manuscript Table 1 from Vassall et al.^6^*

**Figure S2. Model inputs and calibrated model results in open and closed systems**

| A. Number of people by symptom levels |  |
| --- | --- |
|  |  |
| B. Proportion of patients with respective symptom |  |
|  |  |
| C. Number of people who are cured and die |  |
|  |  |
| D. Duration of symptoms |  |
|  |  |

**Table S3. Zambia model input parameters**

These values (monthly transition rates between symptom levels and monthly probabilities of seeking care) were inputted into a Markov model which was constructed to reflect the diagnostic algorithm (CXR and Xpert) used for ACF in the Zambia TB REACH program.

| **Model Parameter** | | **Base Value** | **Distiribution** | **Low Value** | **High Value** | **Source** |
| --- | --- | --- | --- | --- | --- | --- |
| *Disease Epidemiology* | |  |  |  |  |  |
| HIV prevalence | | 0.113 | Beta | 0.1 | 0.13 | 7 |
| TB prevalence in HIV+ patients | | 0.59 | Beta | 0.46 | 0.70 | 3 |
| Proportion of TB cases who have not been treated in the past | | 0.95 | Beta | 0.90 | 1.0 |  |
| TB prevalence in general population | | 0.00346 | Beta | 0.0029 | 0.0043 |  |
| Proportion of TB cases who are MDR, previously treated | | 0.18 | Beta | 0.14 | 0.22 |  |
| Proportion of TB cases who are MDR, treatment-naive | | 0.028 | Beta | 0.025 | 0.031 |  |
| *Efficacy of Diagnostic Tests* | |  |  |  |  |  |
| Probability of high bacterial load on a smear, HIV+ patient | | 0.25 | Beta | 0.25 | 0.44 | 8 |
| Probability of a high bacterial load on a smear, HIV- patient | | 0.65 | Beta | 0.5 | 0.8 |  |
| Smear sensitivity for high bacterial load patient | | 1.0 | Beta | - | - | Estimate |
| Smear sensitivity for low bacterial load patient | | 0.51 | Beta | 0.43 | 0.59 | 9 |
| Sensitivity of Xpert for High Bacterial Loads | | 0.98 | Beta | 0.97 | 0.99 | 10 |
| Sensitivity of Xpert for Low Bacterial Loads | | 0.68 | Beta | 0.59 | 0.75 |  |
| Sensitivity of Xpert for rifampin resistance | | 0.95 | Beta | 0.90 | 0.97 |  |
| Sensitivity of chest X-ray | | 0.90 | Beta | 0.85 | 0.95 | 5 |
| Sensitivity of culture | | 1.0 | Beta | - | - | Estimate |
| Sensitivity of clinical diagnosis (asymptomatic) | | 0 | Beta | - | - |  |
| Sensitivity of clinical diagnosis (mildly symptomatic) | | 0.5 | Beta | 0 | 1.0 |  |
| Sensitivity of clinical diagnosis (strongly symptomatic) | | 1.0 | Beta | 0 | 1.0 |  |
| Specificity of clinical diagnosis (asymptomatic) | | 1.0 | Beta | - | - |  |
| Specificity of clinical diagnosis (mildly symptomatic) | | 0.95 | Beta | 0.90 | 1.0 |  |
| Specificity of clinical diagnosis (strongly symptomatic) | | 0.95 | Beta | 0.90 | 1.0 |  |
| Specificity of Chest X-Ray | | 0.90 | Beta | 0.85 | 0.95 |  |
| Specificity of Sputum Smear Microscopy | | 1.0 | Beta | - | - |  |
| Specificity of Xpert | | 0.98 | Beta | 0.97 | 0.99 | 5 |
| *Monthly Untreated TB Mortality (per 1000 person-years)* | |  |  |  |  |  |
| HIV positive, Smear positive | | 0.06 | Beta | 0.0408 | 0.0799 | 6 |
| HIV positive, Smear negative | | 0.05430 | Beta | 0.0408 | 0.0799 |  |
| HIV negative, Smear positive | | 0.02062 | Beta | 0.0176 | 0.0288 |  |
| HIV negative, Smear negative | | 0.00829 | Beta | 0.0071 | 0.0095 |  |
| *Probability of cure with treatment* | |  |  |  |  |  |
| Cost of first line (category 1) Treatment for HIV positive, MDR | | 0.41 | Beta | 0.35 | 0.46 | 11 |
| Cost of first line (category 1) Treatment for HIV negative, MDR | | 0.61 | Beta | 0.55 | 0.66 |  |
| MDR Treatment for HIV positive, MDR positive, Treatment Naïve | | 0.548 | Beta | 0.50 | 0.65 | 12 |
| MDR Treatment for HIV positive, MDR positive, Previously Treated | | 0.483 | Beta | 0.40 | 0.60 |  |
| MDR Treatment for HIV negative, MDR positive, Treatment Naïve | | 0.748 | Beta | 0.70 | 0.85 |  |
| MDR Treatment for HIV negative, MDR positive, Previously Treated | | 0.683 | Beta | 0.60 | 0.80 |  |
| *TB Epidemiology* | |  |  |  |  |  |
| Proportion of asymptomatic TB patients | | 0.2 | Beta | 0.15 | 0.25 | Symptom transition model |
| Proportion of nonspecific TB patients | | 0.3 | Beta | 0.25 | 0.35 |  |
| Proportion of classic TB patients | | 0.5 | Beta | 0.45 | 0.55 |  |
| *Connection to Health Care System* | |  |  |  |  |  |
| Proportion of symptomatic (mild/strong) patients who attend ACF | | 0.86 | Beta | 0.5 | 1.0 | Symptom transition model |
| Monthly probability of a patient passively contacting the health system for TB diagnostics | Asymptomatic | 0 | Beta | 0 | 0 |  |
|  | Nonspecific | 0.2 | Beta | 0 | 0.5 |  |
|  | Classic | 0.4 | Beta | 0.2 | 0.6 |  |
| *Monthly Symptom Level Transition Rate* | |  |  |  |  |  |
| Probability of transition from no symptom to cure | | 0.05 | Beta | 0.04 | 0.06 | Symptom transition model |
| Probability of transition from mild symptom to no symptom | | 0.12 | Beta | 0.1 | 0.2 |  |
| Probability of transition from no symptom to mild symptom | | 0.24 | Beta | 0.2 | 0.3 |  |
| Probability of transition from mild symptom to strong symptom | | 0.80 | Beta | 0.7 | 0.9 |  |
| Probability of transition from strong symptom to mild symptom | | 0.40 | Beta | 0.3 | 0.8 |  |
| Probability of transition from strong symptom to death | | 0.05 | Beta | 0.4 | 0.6 |  |
| *Cost Parameters (2018 USD)* | |  |  |  |  |  |
| Cost of screening for ACF day | | $3.33 | Gamma | 1.53 | 5.79 | Figure A1 and Table 2 |
| Cost of screening at clinic (status quo) | | $6.86 | Gamma | 5.83 | 7.89 |  |
| Cost of mobile Chest Xray test | | $13.31 | Gamma | 7.54 | 25.62 |  |
| Cost of Xpert test | | $16.34 | Gamma | 9.68 | 27.89 |  |
| Cost of first line (category 1) treatment | | $354 | Gamma | 300 | 407 | 13 |
| Cost of MDR treatment | | $3,407 | Gamma | 2,896 | 3,919 |  |

**Figure S3. Markov symptom-based care seeking model**

100,000 individuals defined by TB/HIV status and symptom level and modeled as having a one-time chance to attend ACF or routine care based on a monthly probability of accessing care throughout the duration of the analysis. Passive contact with the health system via routine care resulted in a chance for diagnosis via sputum smear microscopy and initiation of first line treatment, based on symptom level. Individuals with untreated TB at the end of each monthly cycle experienced a monthly probability of symptom level transition (progression or regression).

| 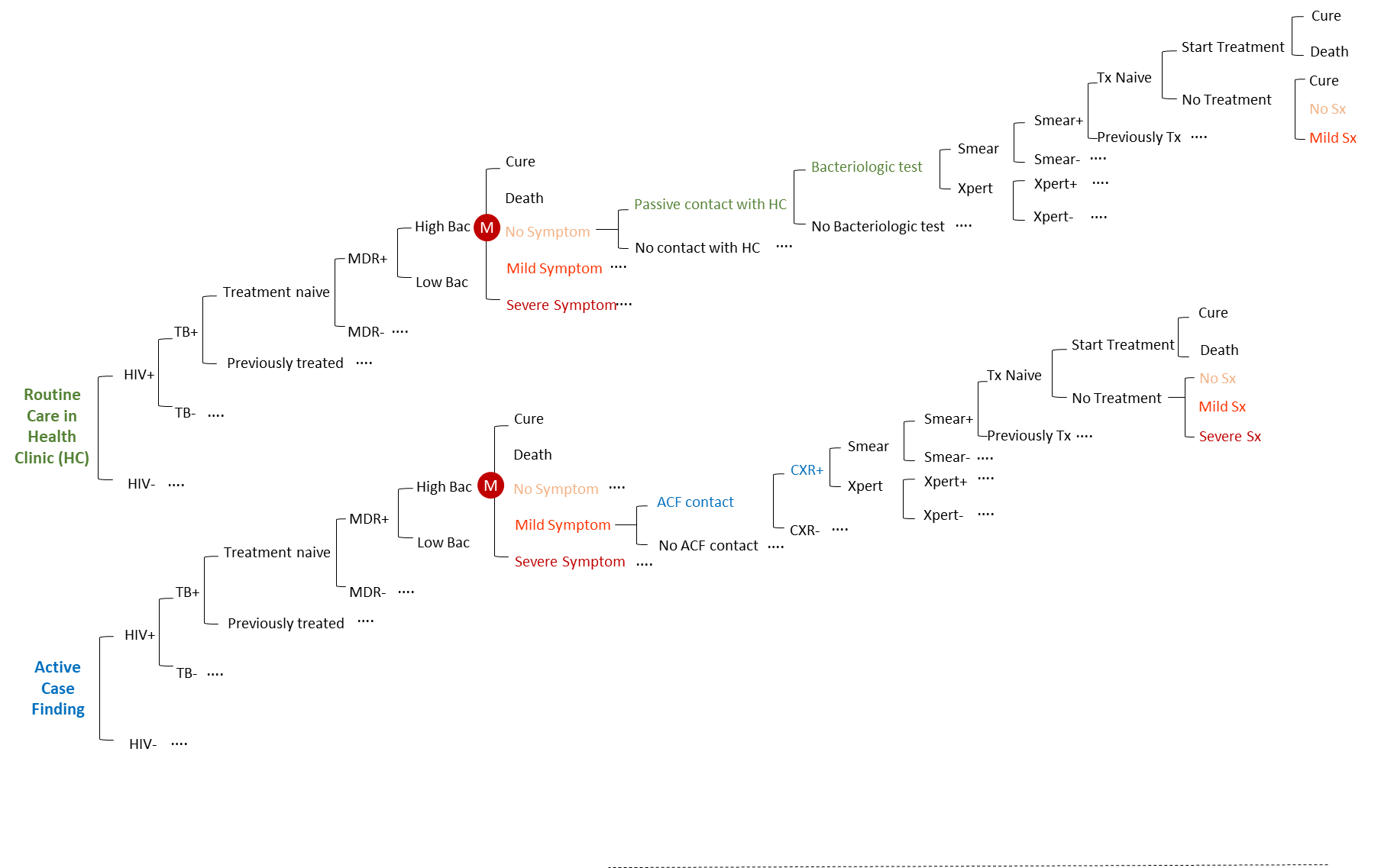 |
| --- |

**Figure S4. Incremental number of diagnoses and deaths averted under active tuberculosis case finding intervention compared to routine care over a Five Year Horizon, per 100,000 population**

The y-axis in each panel displays incremental number of diagnoses and TB deaths averted by a one-off comprehensive active case finding (ACF) intervention compared to the status quo (passive case finding), over the sixty-months period post implementation of the ACF intervention. The incremental number of diagnoses (black line) under ACF compared to the status quo are large at the onset but subsequently decreasing over time but number of deaths averted (grey line) increases over time. In a scenario of the probability of people passively seeking care is high (60%), the incremental number of diagnoses (black long dashed line) and deaths averted (grey long dashed line) are about 300 per 100,000 at 16^th^ month. In a scenario of the probability of people see care to the status quo is low (20%), the incremental number of diagnoses (black short dashed line) and deaths averted (grey short dashed line) are about 750 per 100,000 at 28^th^ month.

**References**

1. Johnson K, Uhlig E, Jo Y, Creswell J, Qin Z, Dowdy D, Sohn H. Incremental Value of Tuberculosis Active Case Finding in the Context of Symptom-Associated Care Seeking. In review.

2. Tiemersma EW, van der Werf MJ, Borgdorff MW, Williams BG, Nagelkerke NJ. Natural history of tuberculosis: duration and fatality of untreated pulmonary tuberculosis in HIV negative patients: a systematic review. *PLoS One.* 2011;6(4):e17601.

3. World Health Organization. Global Tuberculosis Report. Zambia Tuberculosis Profile 2018. <https://www.who.int/tb/publications/global_report/en/>

4. Storla DG, Yimer S, Bjune GA. A systematic review of delay in the diagnosis and treatment of tuberculosis. *BMC Public Health.* 2008;8:15.

5. Langendam MW, Akl EA, Dahm P, Glasziou P, Guyatt G, Schunemann HJ. Assessing and presenting summaries of evidence in Cochrane Reviews. *Syst Rev.* 2013;2:81.

6. Vassall A, van Kampen S, Sohn H, et al. Rapid diagnosis of tuberculosis with the Xpert MTB/RIF assay in high burden countries: a cost-effectiveness analysis. *PLoS Med.* 2011;8(11):e1001120.

7. UNAIDS. Zambia. <https://www.unaids.org/en/regionscountries/countries/zambia>

8. Salje H, Andrews JR, Deo S, et al. The importance of implementation strategy in scaling up Xpert MTB/RIF for diagnosis of tuberculosis in the Indian health-care system: a transmission model. *PLoS Med.* 2014;11(7):e1001674.

9. Cattamanchi A, Dowdy DW, Davis JL, et al. Sensitivity of direct versus concentrated sputum smear microscopy in HIV-infected patients suspected of having pulmonary tuberculosis. *BMC Infect Dis.* 2009;9:53.

10. Horne DJ, Kohli M, Zifodya JS, et al. Xpert MTB/RIF and Xpert MTB/RIF Ultra for pulmonary tuberculosis and rifampicin resistance in adults. *Cochrane Database Syst Rev.* 2019;6:CD009593.

11. Espinal MA, Kim SJ, Suarez PG, et al. Standard short-course chemotherapy for drug-resistant tuberculosis: treatment outcomes in 6 countries. *JAMA.* 2000;283(19):2537-2545.

12. Isaakidis P, Casas EC, Das M, Tseretopoulou X, Ntzani EE, Ford N. Treatment outcomes for HIV and MDR-TB co-infected adults and children: systematic review and meta-analysis. *Int J Tuberc Lung Dis.* 2015;19(8):969-978.

13. Yadav RP, Nishikiori N, Satha P, Eang MT, Lubell Y. Cost-effectiveness of a tuberculosis active case finding program targeting household and neighborhood contacts in Cambodia. *Am J Trop Med Hyg.* 2014;90(5):866-872.
